# Supplementary material for: Impact of phage enrichment on the observed infant gut phageome
Source: Microbiol Spectr. 2026 Mar 24;14(5):e02153-25. doi: 10.1128/spectrum.02153-25 (PMC13141913; doi:10.1128/spectrum.02153-25)
Supplement: Supplemental figures — Figures S1 to S4. [file spectrum.02153-25-s0001.pdf]

Supplementary figures

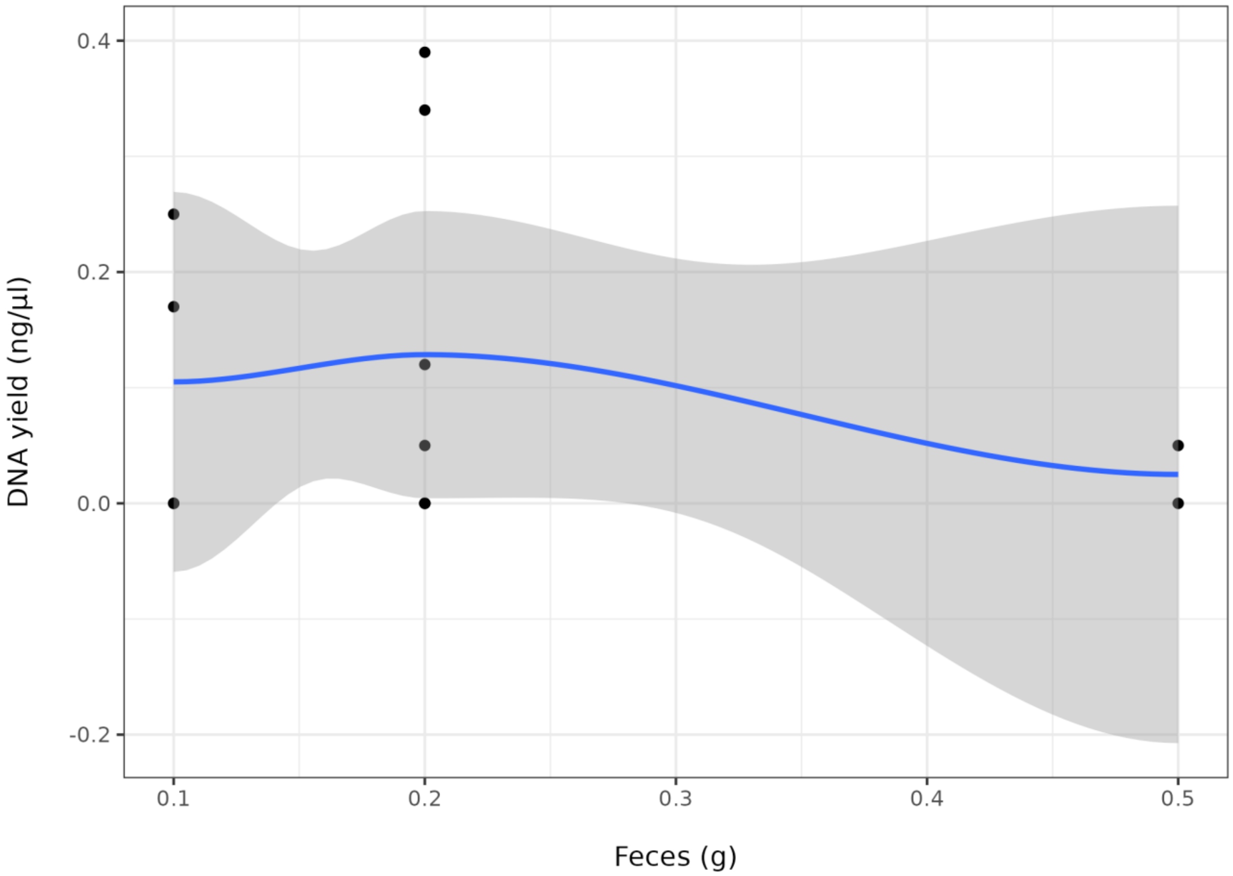

Figure S1. Correlation between DNA yield and the amount of faecal material (g) used in Protocol D1 for input standardization. The blue line represents the linear regression fitted with the geom_smooth() function in R, and the shaded grey area indicates the confidence interval.

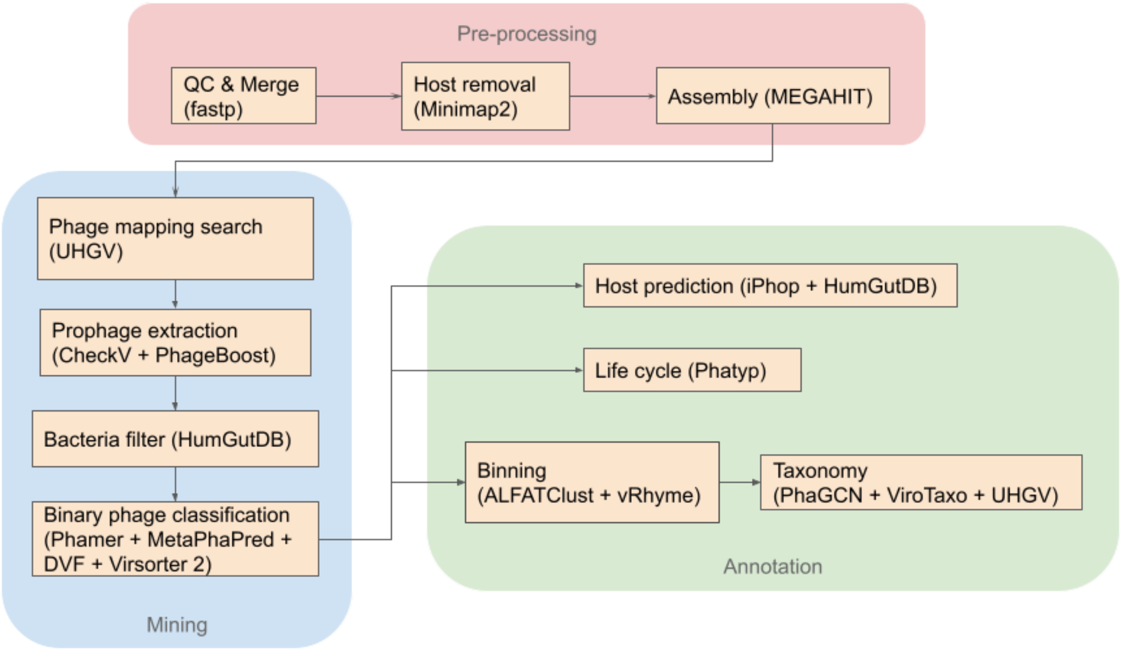


```
fastp -i $r1 -I $r2 --merge --include_unmerged --merged_out "${sname}.fq.gz" -h ${sname}.html -j ${sname}.json -l 50 -q 20 -n 0 -w 8 -f 2 -t 2 -F 2 -T 2 --dedup --dup_calc_accuracy 3 --overlap_len_require 20 --fix_mgi_id --detect_adapter_for_pe --correction --overlap_diff_limit 3

minimap2 -t 8 -u both --split-prefix=${sname}.tmp -a $new/software/minimap2DB/human_shortread.mmi $r3 | samtools view -h -f 4 |

samtools sort -n | samtools view -bh > ${sname}_nohost.bam samtools fastq -n ${sname}_nohost.bam > ${sname}.fq

megahit --presets meta-sensitive -r all.fq.gz -t $c -m 400e9 -o final
```

```
blastn -query ../${sname}.fna -db $UHGVDB -num_threads $c -evalue 0.001 -perc_identity 70 -out ${sname}.tsv -qcov_hsp_perc 70 -outfmt "6 qseqid sseqid pident qlen slen length"

checkv end_to_end ${sname}_after_blast.fna ${sname}_checkv -t 4 --remove_tmp

PhageBoost -f ${sname}_after_checkv.fna -j $c -o ${sname}_phageboost -meta 0 -t $fscore

kraken2 --confidence 0.3 --db $humgutdb --report /dev/null --output ${sname}.kreads ${sname}_excluded.fna
```

```
python $mpp_home/predict.py -i $ff -o ${ff}_mpp.tsv #Metaphapred

phabox2 --task phamer --dbdir $PDIR/phabox_db_v2 --contigs $inputF --threads $c --midfolder ${sname}_tmpphamer --outpth ${sname}_phamer --len $lfilt

python $SDIR/DeepVirFinder/dvf.py -i $inputF -m $SDIR/DeepVirFinder/models  -o . -l $lfilt -c $c

virsorter run -w vs2_${ff} -i $ff  --min-length 2000 -j 4 --min-score 0.9 --high-confidence-only --provirus-off --rm-tmpdir all &
```

```
iphop predict -d /scratch/project_2007362/software/iphop_db/Aug_2023_pub_rw -t $cpu -o ${sname_assembly}_${tmpname}_iphop -f ${ff} --no_qc &

vRhyme -i tmp_${sname_assembly}.fna -b $bamf/*_map.bam -t $c -o ${sname_assembly}_vrhyme

$new/software/ALFATClust/main/alfatclust.py -i $virfasta -o ${sname}_f.atclust -e ${sname}_f.repatclust -t $c -b dna --seed 1 -l 0.7

phabox2 --task phagcn --dbdir $PDIR/phabox_db_v2 --contigs $virfasta --threads $c --midfolder ${sname_assembly}_tmpgcn --outpth ${sname_assembly}_phagcn --reject 0.5 --len $lfilt

phabox2 --task phatyp --dbdir $PDIR/phabox_db_v2 --contigs $virfasta --threads $c --midfolder ${sname_assembly}_tmptyp --outpth ${sname_assembly}_phatyp --reject 0.5 --len $lfilt

blastn -query $vircontigs -db $UHGVDB -num_threads 2 -evalue 0.001 -perc_identity 70 -out ${sname_assembly}.tsv -qcov_hsp_perc 70 -outfmt "6 qseqid sseqid pident qlen slen length"

python $SDIR/predict.py --model_path $SDIR/uhgv_genus_mq.pkl --seq ${home_project}/2_assembly/$vircontigs > ${sname_assembly}_virustaxo.tsv # ViroTaxo

command for pretraining ViroTaxo using UHGV: python build.py --meta uhgv_mq_plus/gdata.csv --se uhgv_mq_plus/genus.fna --k 27 --saving_path uhgv_genus_mq.pkl
```

Figure S2. Bioinformatic protocol with focus on phage identification and quantification starting from raw reads, three rounds of phage classification, double binning, and different annotation levels (host, taxonomy, lifecycle and phage activity). Parameters for each step

are listed below in their corresponding stage color and whole scripts used can be found in the supplementary materials slurm_scripts.

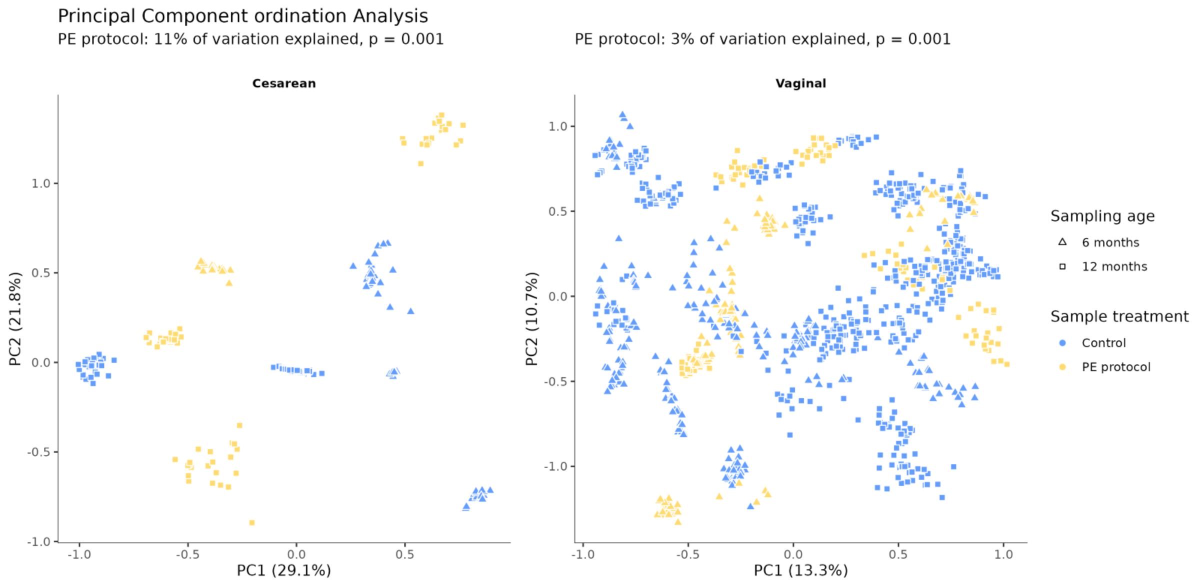

Figure S3: Principal coordinates analysis of samples (using Pearson distance). Analysis was performed using the log-transformed relative units of phages at family. Blue points represent control samples while yellow points are protocol treated samples. Triangle shaped points represent 6-months-old infant samples and square points indicate 12-months-old samples.

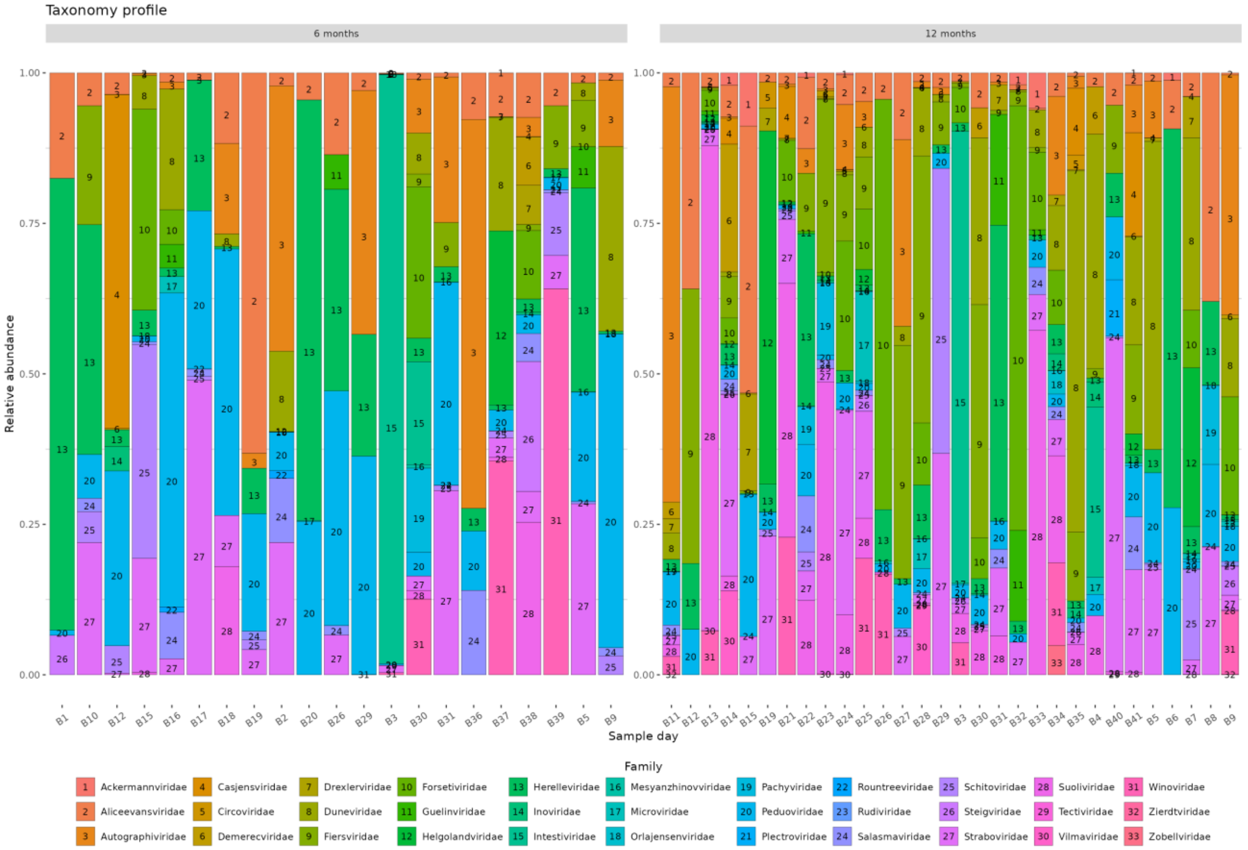

Figure S4: Taxonomic profiles by Baby ID and age. The profiles were generated by averaging the daily phageome at family level for each infant and rescaling the values from 0 to 1 for better visualization. Additionally, phage family ID numbers were assigned to help track the corresponding colors of each phage family.
